# Medical Hall for Baltimore

**Published:** 1880-02

**Authors:** 


### EDITORIAL DEPARTMENT.

A MEDICAL HALL FOR BALTIMORE.—The question of a central hall or building in Baltimore suitable for and devoted exclusively to the use of the medical profession, has recently taken definite shape, and an association has already been organized for the purpose of carrying it into execution.

A careful consideration of our present circumstances, accommodations and needs, convinces us, and we feel sure will convince all who sincerely desire the advancement of professional interests among us, of the existence of such a necessity.

In a great and rapidly growing city like this, with many of the features of a metropolis, with important intellectual, commercial, manufacturing and other interests centreing in it, and drawing from all quarters so many thousands of visitors in search of pleasure, profit, or improvement, it would seem to be a plain duty to provide in every way for the hospitable reception and entertainment of our guests, and such an obligation is incumbent upon us as a profession no less than as citizens. Besides, the mutual benefits that would accrue to both guests and host are not hard to conceive of nor inconsiderable.

Furthermore, looking at our own wants, does it not seem as though six hundred physicians would need some central establishment, some building suitable for society and committee meetings, for a library (of which we have a very good nucleus), museum, etc.? At present our accommodations are utterly inadequate to our wants; what expectation of improvement, then, can we indulge for the future when these wants are daily growing greater? While we believe that those in charge have done the best that is possible under present arrangements, we can expect no great advance until we have absolute control of a building in which we will feel all the interest of ownership, and where suitable provision can be made for instituting or rendering more efficient all those instrumentalities which are calculated to secure to us our rights, individual and collective, to enlighten us as to our duties, and to make us better members of our profession and of society.

See what the New York Academy of Medicine has accomplished; see its plendid new hall and library of over 10,000 volumes. Ought not such an example to prove a powerful incentive to us and teach us what we can do by combined effort, aided by individual generosity?

Cannot some one be found in our midst, able and willing, like Du Bois, to make a donation of money or ground toward this most commendable enterprise? Such a gift would doubtless react on others and give a powerful impulse to the undertaking.

We urge the wealthier members of the profession seriously to consider whether they can make a wiser disposition of their means than by donating to the profession a site for their building in some central and judiciously chosen locality. Is there any better way to deserve the thanks of contemporaries and to hand down one's name to be cherished by a grateful posterity? And, on the other hand, could not the profession well afford to commemorate such an act by connecting the name of the benefactor with a building which would be so largely due to his generosity and public spirit?

